# Individual Preferences and Social Interactions Determine the Aggregation of Woodlice

**DOI:** 10.1371/journal.pone.0017389

**Published:** 2011-02-25

**Authors:** Cédric Devigne, Pierre Broly, Jean-Louis Deneubourg

**Affiliations:** 1 Université Lille Nord de France, Lille, France; 2 UCLille, FLST, Laboratoire Environnement and Santé, Lille, France; 3 Université libre de Bruxelles, Campus de la Plaine, Bruxelles, Belgium; University of Bristol, United Kingdom

## Abstract

**Background:**

The aggregation of woodlice in dark and moist places is considered an adaptation to land life and most studies are focused on its functionality or on the behavioural mechanisms related to the individual's response to abiotic factors. Until now, no clear experimental demonstration was available about aggregation resulting from inter-attraction between conspecifics.

**Methodology/Main Findings:**

We present the dynamics of aggregation, not previously described in detail in literature, as being independent of the experimental conditions: homogeneous and heterogeneous environments with identical or different shelters. Indeed whatever these conditions, the aggregation is very quick. In less than 10 minutes more than 50% of woodlice were aggregated in several small groups in the homogeneous environment or under shelters in the heterogeneous environment. After this fast aggregation, woodlice progressively moved into a single aggregate or under one shelter.

**Conclusions/Significance:**

Here we show for the first time that aggregation in woodlice implies a strong social component and results from a trade-off between individual preferences and inter-attraction between individuals. Moreover, our results reveal that the response to the heterogeneities affects only the location of the aggregates and not the level of aggregation, and demonstrate the strong inter-attraction between conspecifics which can outweigh individual preferences. This inter-attraction can lead to situations that could seem sub-optimal.

## Introduction

There are about 10,000 described species of isopods [1], and nearly half of them are terrestrial and belong to the suborder Oniscidae [2]. Woodlice have a great ecological role in the decomposition process due to their digestive capabilities [3]. Furthermore, woodlice also participate in the dispersal of microbiota by voiding faecal pellets. Their importance in the soil ecology and their physiology makes woodlice potentially useful as a bioindicator for detecting and monitoring bio-accumulation of heavy metals [4]–[6]. Due to the key roles of woodlice in soil ecosystems and in the spread of various microbiotic populations, it is necessary to better understand the aggregation patterns of such organisms from an evolutionary-ecological perspective. Aggregation is one of the most basic social phenomena and is a proximal prerequisite for the development of other forms of cooperation such as the use of public information about the quality of environmental resources [7]–[10]. In this respect, it may control different density dependent processes and may influence the dynamics of population at large spatio-temporal scale [11]. Isopods are an ideal model system for the study of these questions; however, surprisingly many gaps in our knowledge still remain and therefore we do not yet truly appreciate the extent of the consequences of aggregation for the physiology, behaviour, or evolution of species [12], [13].

In the crustaceans, the suborder Oniscidea consists of terrestrial families only [14]. In this respect, woodlice have been intensively studied to understand their adaptation to land life [15]–[17]. The adaptations can be structural [18], physiological [3], [19], [20], or behavioural [21]. Most of the behavioural adaptations described in the literature concern the individual response to environmental parameters (and interactions between them) [15], [21]–[23]. For example, it has been shown that orientation to light changes from positive to negative with the transition from the sea to the littoral zone in *Ligia*, and that this is coherent with the search for dark, moist, and cool places [15], [21]. However, some behavioural adaptations are related to groups of individuals. In this respect, aggregation of woodlice is a well known phenomenon which is at the origin of the theory of the Allee effect [24], [13], [25], [26]. The increase in density of woodlice in a location enhances their survival in harsh conditions by reducing water losses [24], [27] and this gregarious behaviour is considered an adaptation to terrestrial life [28]–[30]. It is important to note that aggregation is observed in numerous crustaceans [17], for example, in the aquatic isopods *Asellus communis* (as a reaction to running water, *Asellus* seek quiet water areas; [31]) or *Lirceus fontinalis* (as a reaction to harsh conditions such as drought and high temperature; [32], [33]).

At its most basic, aggregation is just a grouping of animals [34]. In this case, aggregation results from a response to local environmental heterogeneities that only imply tolerance between individuals [12], [35]–[37]. Another mechanism of aggregation is that resulting from inter-attraction between conspecifics which defines gregarious species [38], [39]. Very little information is available on the inter-attraction between woodlouse individuals. However some experiments show that aggregative pheromones are at work. Binary choice studies carried out in an olfactometer (Y-maze) show that olfaction could permit woodlice to find conspecifics [40], [3]. Indeed, in the Y-maze, the focal individuals were more likely to approach the group of 50 woodlice when given the choice between the group and nothing [41], [42]. Moreover, experiments of substrate marking also suggest the existence of an aggregation pheromone in the faeces. Indeed papers marked with faeces were preferred to unmarked papers in binary choice tests carried out in Petri dishes. However, the chemical nature of the pheromone is still unknown [43]. Hence, more ethological tests are necessary to better understand the implications of chemical communication in the aggregation process.

Except for brief descriptions carried out by Farr and by Takeda [42], [44] and an observation in a homogeneous environment by Allee [12], the aggregation dynamics and the resulting patterns have not been studied until now. No information about the kinetics of the aggregation process, the influence of environmental conditions on these kinetics, or the morphology of aggregates is available in literature. This information is important because it is well known that observation of attraction between congeners (e.g. an olfactometric test showing that olfaction permits woodlice to find conspecifics) is not sufficient to draw conclusions about patterns of aggregation and the stability of aggregates [45], [46]. In groups of living organisms, the spatiotemporal distribution of the population results from the synergy between the individual preferences and the inter-attraction between conspecifics. The objective of this paper is to show that in woodlice, inter-attraction is at work, and how its synergy with individual preferences governs the dynamics and the patterns of aggregation.

## Materials and Methods

### The species

The common woodlouse *Porcellio scaber* Latreille, 1804 is a widely distributed terrestrial isopod (Isopoda: Porcellionidae) well known to form aggregates. There is substantial information about individual preferences of *Porcellio scaber* and hence this species is a good model for study of the aggregation mechanisms [17], [20], [23], [24].

Woodlice were collected in the gardens of Lille Catholic University (northern France). They were reared in terraria (410×240×225 mm) at the bottom of which a plaster layer, regularly moistened, kept the humidity at 75±10%. A litter of maple, beech, and oak leaves also formed their food resources. In addition, bark was provided to offer shelters for woodlice. Room temperature was kept at 23±2°C. Terraria were maintained at a photoperiod of 14∶10 (L:D).

### Experimental set up

**Figure 1:**
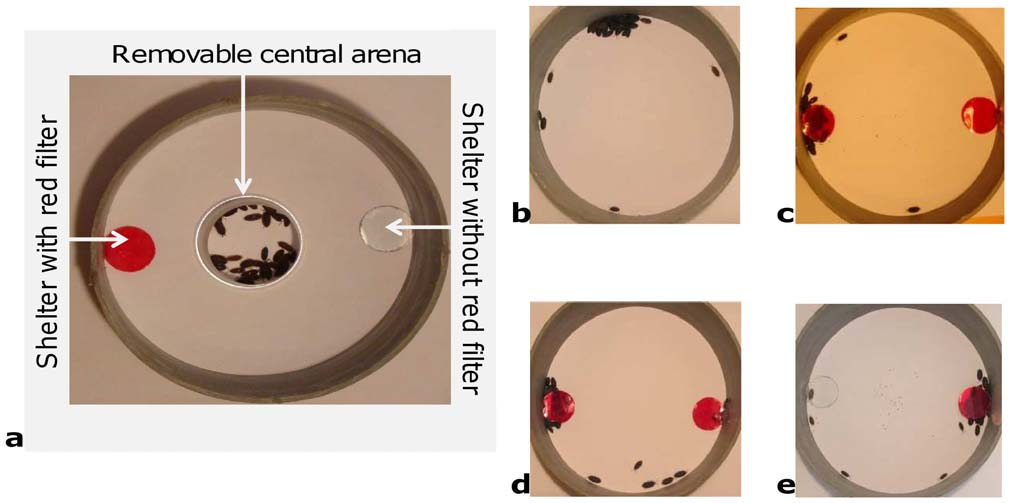
Experimental set up. **a**: with small removable central arena; **b**: in homogeneous environment; **c**, **d**, and **e**: in heterogeneous environment with two identical shelters (c), with two shelters, one with one layer of red filter and the other with two layers of red filter (d), and with two shelters, one without a filter and the other with one layer of red filter (e).

The basic experimental set up consisted of a homogeneous arena (PVC tube, 193 mm in diameter) with a small removable central arena (65 mm of diameter) where woodlice were placed before the beginning of the experiment in order to calm them down (cf. Figure 1a,b).

In the homogeneous set up, three light intensities were tested: ***A:*** Low Brightness (0 lux) with no light bulb, where the experimental set up was shut in a cardboard box with only one opening on the top to allow the video recording, ***B:*** medium brightness (166 lux) obtained with a 40 W bulb, ***C:*** high brightness (1069 lux) obtained with a 60 W light bulb.

Experiments in a heterogeneous environment were also carried out, where two shelters (small glass plate - 35 mm in diameter) were added to the arena previously described (cf. Figure 1c-e).

In the heterogeneous set up, the arena brightness was medium (166 lux, 40 W light bulb). Hence three light intensities could be obtained under the shelters: 166 lux when there was no filter, 56 lux when the shelter was covered by one layer of ROSCO® filters (ref. Roscolux #19 Fire – this filter also changed the spectrum of light by transmitted nearly only red energy) and 41 lux when it was covered by two layers.

Three binary choices conditions were tested: ***D:*** one shelter with No filter vs. one shelter with 1 filter, ***E:*** 1 filter vs. 2 filters and ***F:*** 2 filters vs. 2 filters.

To summarize, there were two conditions with two different shelters (C and D) and one condition with two identical shelters (F).

The light bulbs were placed at 80 cm above the experimental arena to prevent over-heating. The light intensity was measured with a digital lux meter (MS-1300 – Voltcraft®) (results are shown in Table 1).

**Table 1:** Description of experiments carried out and results about aggregation.

Type of set up	Light intensity	Reference of experiments	Percentage of experiments where at least one aggregate is observed	Percentage of experiments with aggregation where one of the shelters is chosen	For experiments where one of the shelters is chosen: Percentage of experiments where the darkest shelter is chosen
**Homogeneous environment**	0 lux	A	100 (N = 20)	-	-
	40 W light bulb (166 lux)	B	100 (N = 20)	-	-
	60 W light bulb (1069 lux)	C	90 (N = 20)	-	-
**Heterogeneous environment**	None vs 1 red filter (166 vs 56 lux)	D	100 (N = 24)	75 (N = 24)	88.9 (N = 18)
	1 vs 2 red filters(56 vs 41 lux)	E	100 (N = 36)	91.7 (N = 36)	48.5 (N = 33)
	2 vs 2 red filters (41 vs 41 lux)	F (control)	96.7 (N = 30)	70 (N = 30)	(right) 57.1 (N = 21)
		*χ* ***^2^ test***	***–***	***NS*** *χ* ***^2^*** = ***5.31 (df = 2)***	***D≠E,F: p<0.05*** *χ* ***^2^*** = ***8.19 (df = 2)***

Selection was determined if the distribution of woodlice between both shelters was significantly different from an equal repartition of woodlice between these shelters (binomial test). The χ^2^ test determined differences between proportions.

The experimental set up was placed on a white sheet of paper which was changed between each experiment.

Woodlice were considered to be aggregated when they were at a distance from their neighbours less than or equal to the average length of a woodlouse (0.5 cm). Moreover, groups were only considered to be aggregates when they were stable (i.e. in the same location) for 3 minutes.

Here are definitions of the terminology used in this document hereafter:

#### Dark shelter

Shelter with one or two red filters (56 or 41 lux, respectively).

#### Bright shelter

Shelter without a red filter (166 lux).

#### Total population of aggregated woodlice

Total number of woodlice aggregated, possibly in several aggregates.

#### Final aggregate

To define the final aggregate, the size and location of the bigger aggregate at the end of the experiment were assessed. The dynamic of the final aggregate corresponds to the change of number of woodlice at this location.

#### Secondary aggregates

Small aggregates which appeared during the experiment and may progressively disappear (or not). These secondary aggregates can coexist with the bigger final aggregate.

### Experimental procedure

Forty woodlice were placed in the small central arena and left there for 5 minutes to settle down. Then, the experiments began by removing the central arena, releasing woodlice, which travelled toward the edge of arena. Each experiment was video recorded for 45 minutes (Video S1).

To avoid any bias, dark and bright shelters were located equally either at the right or left of the set up. Our F condition, with two identical shelters, allowed us to ensure that there was no skew in our experimental set up since the right and left shelters were chosen equally frequently (Table 1; 57.1% vs 42.9%, respectively, N = 21; Fisher's exact test, p>0.05).

### Data analysis

In the homogeneous environment, in order to check if our distribution of woodlice in each experiment corresponded to aggregation, we analysed radial and angular distributions of woodlice. Kolmogorov-Smirnov goodness of fit tests were used to compare, for each experiment, the observed radial distribution with a simulated uniform distribution. Rayleigh tests were used to describe angular distributions. The coupling of the two tests allowed us to describe our observed distribution as being an aggregation (cf. Text S1, Figures S1 and S2).

In the heterogeneous environment, to determine whether woodlice selected one shelter preferentially, binomial tests were carried out with H_o_ assuming an equal distribution of woodlice between both shelters. The “winning” shelter was the shelter with the bigger aggregate at the end of the experiment and the “losing” shelter was the other one [47], [48].

## Results

### Homogeneous environment (conditions A, B, and C)

#### Is there aggregation?

Significant differences were observed between theoretical uniform distribution and distributions of woodlice observed in experiments for each of the brightness conditions both for radial distance and angular distribution. In terms of the radial distance, all experiments but one showed a distribution significantly different from uniformity (K-S test, D>0.53, p<0.001 for each experiment but one where D = 0.47, p = 0.06). Hence, at the end of the experiments, more than 90% of woodlice were observed at the periphery of the arena (2165 over 2400 woodlice were found at a distance less than 1.5 cm from the periphery of arena).

The radial distribution showed that thigmotaxis is strong in woodlice however this analysis does not provide any information about aggregation due to social effects. Hence, the analysis of the angular distribution was necessary to assess this. The angular distribution observed in experiments also significantly differed from the theoretical uniform distribution (Rayleigh's test, z>3.3, p<0.05 for each of the 60 experiments except for 7 experiments where z<1.85, p>0.05). These results confirmed that woodlice were really aggregated at the periphery of the homogeneous set up whatever the light intensity. At the end of the experiments, most of the individuals were together in a large and stable aggregate.

**Figure 2:**
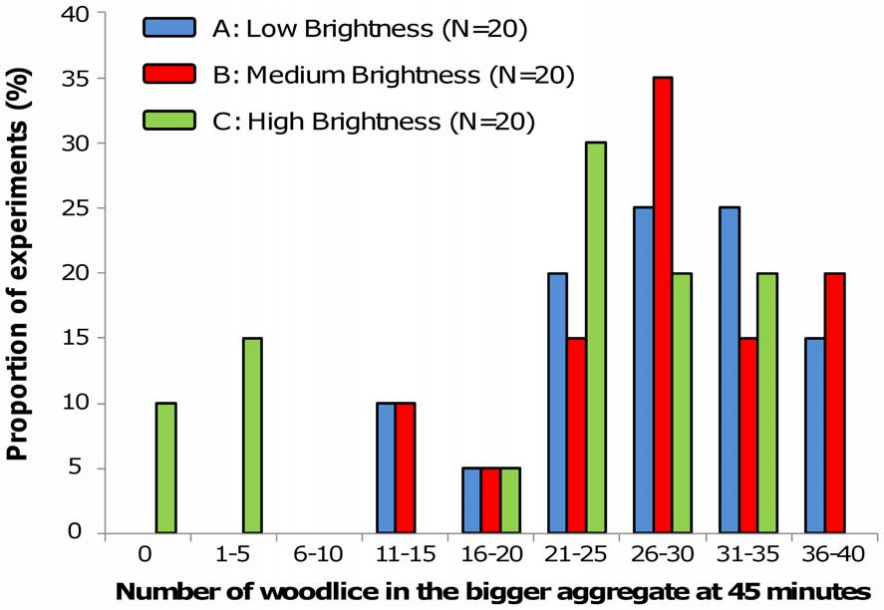
Distribution of woodlice in the final aggregate after 45 minutes in homogeneous set ups.

However, in high brightness, 25% of experiments (N = 20) did not show stable aggregate or had only a small number (less than 10 woodlice) of woodlice in the final aggregate (Figure 2). By contrast experiments with less than 10 woodlice in the final aggregate were never observed in low and medium brightness (Figure 2). Nevertheless, whatever the light intensity, more than 70% of experiments showed a final aggregate containing more than 50% of the whole woodlice population at the end of the experiments (Figure 2; 85%, 85%, and 70% of experiments in low, medium, and high brightness, respectively).

#### Dynamics of aggregation

**Figure 3:**
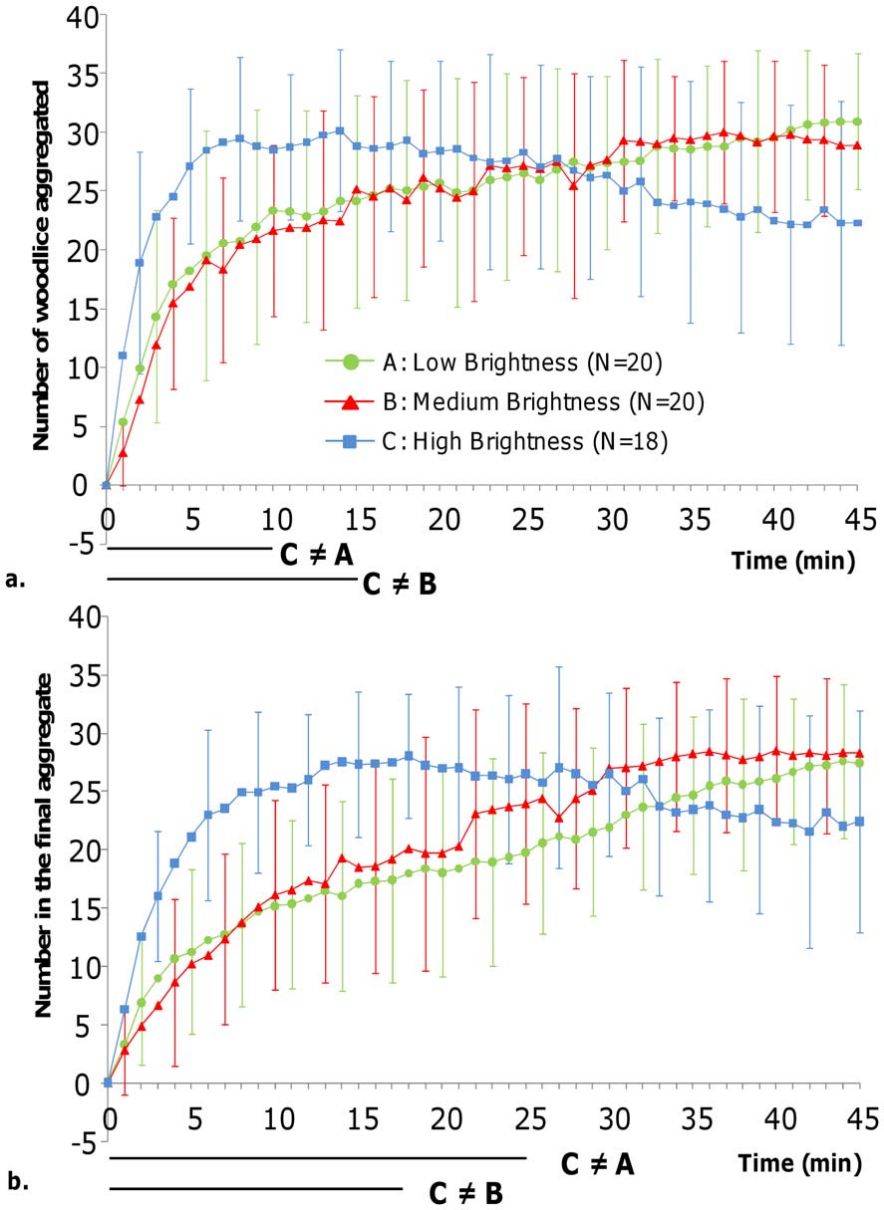
Comparison of dynamics of aggregation in homogeneous set ups. Dynamics were observed in arenas under low, medium, or high brightness corresponding to experimental conditions A, B, and C. **a**: total population of aggregated woodlice; **b**: woodlice aggregated in the final aggregate. Standard deviations are presented for each 3 minutes. The bottom part of the graphic represents the statistical differences obtained minute per minute using Dunn's test, p<0.05: lines show differences.

Whatever the experimental conditions, aggregation was very quick; more than 50% of the woodlice were observed in an aggregate in less than 10 minutes (Figure 3a). The main difference in aggregation dynamics occurred between experiments under high and the two others brightness settings. Indeed, in the first 15 minutes, global aggregation was faster in high brightness than in medium or low brightness (Figure 3a; Kruskal-Wallis test, KW values>6.66, p<0.05 followed by Dunn's test: C≠B and C≠A, p < 0.05). After 15 minutes, no differences were found except at the end of experiments where the total population of aggregated woodlice was significantly lower under high brightness than in the two other conditions (Figure 3a; Kruskal-Wallis test, KW values>6.66, p<0.05 followed by Dunn's test, p<0.05 in the final 5 minutes). Hence, in high brightness, after reaching a maximum very quickly, the number of aggregated woodlice progressively decreased during the experiments (Figure 3a; comparison between 10, 30, and 45 minutes; Friedman's test, Fr = 11.68, df = 2, p<0.01). By contrast, in low and medium brightness, after a rapid increase in the first 10 minutes, this number slowly but significantly continued to increase until the end of the experiments (Figure 3a; comparison between 10, 30, and 45 minutes; Friedman's test, Fr = 21.12 and 10.49, df = 2 for low and medium brightness respectively, p<0.01).

Similar observations could also be made regarding the dynamic of the population in the final aggregate (Figure 3b). Aggregation in the final aggregate was also faster in high brightness than in the other two brightness settings. Indeed, at 10 minutes, 60% of woodlice were already aggregated in high brightness compared to 35% in medium and low brightness (Figure 3b; χ^2^ test, df = 2, χ^2^ = 6.79, p = 0.034). Furthermore, in the first 20 minutes, the number of woodlice in the final aggregate was significantly higher in high brightness (Figure 3b comparisons of average numbers of woodlice in the final aggregates minute per minute were tested by Kruskal-Wallis'test, KW>6.6, p<0.05 followed by Dunn's test, p<0.05).

After 20 minutes of experiments, in high brightness, the number of woodlice in the final aggregate slightly decreased to stabilize at around 20 woodlice. However, the high variability of results did not permit any statistical differences to be observed (Figure 3b; comparison between 10, 30, and 45 minutes in high brightness; Friedman test, Fr = 3.937, p = 0.14). By contrast, under low and medium brightness, the number of woodlice in the final aggregate progressively increased during 45 minutes to reach nearly 30 woodlice at the end of the experiments (Figure 3b; comparison between 10, 30, and 45 minutes. 10≠30, 10≠45 and 30≠45 for low brightness and 10≠30 and10≠45 for medium brightness; Kruskal-Wallis'test KW >25, p<0.001, followed by a Dunn's test, p<0.001).

At the end of the experiments no difference was found between experimental conditions in the average number of woodlice in the final aggregate (Figure 3b; comparison between low, medium, and high brightness at 45 minutes; Kruskal-Wallis test, KW = 3.93, p = 0.14).

**Figure 4:**
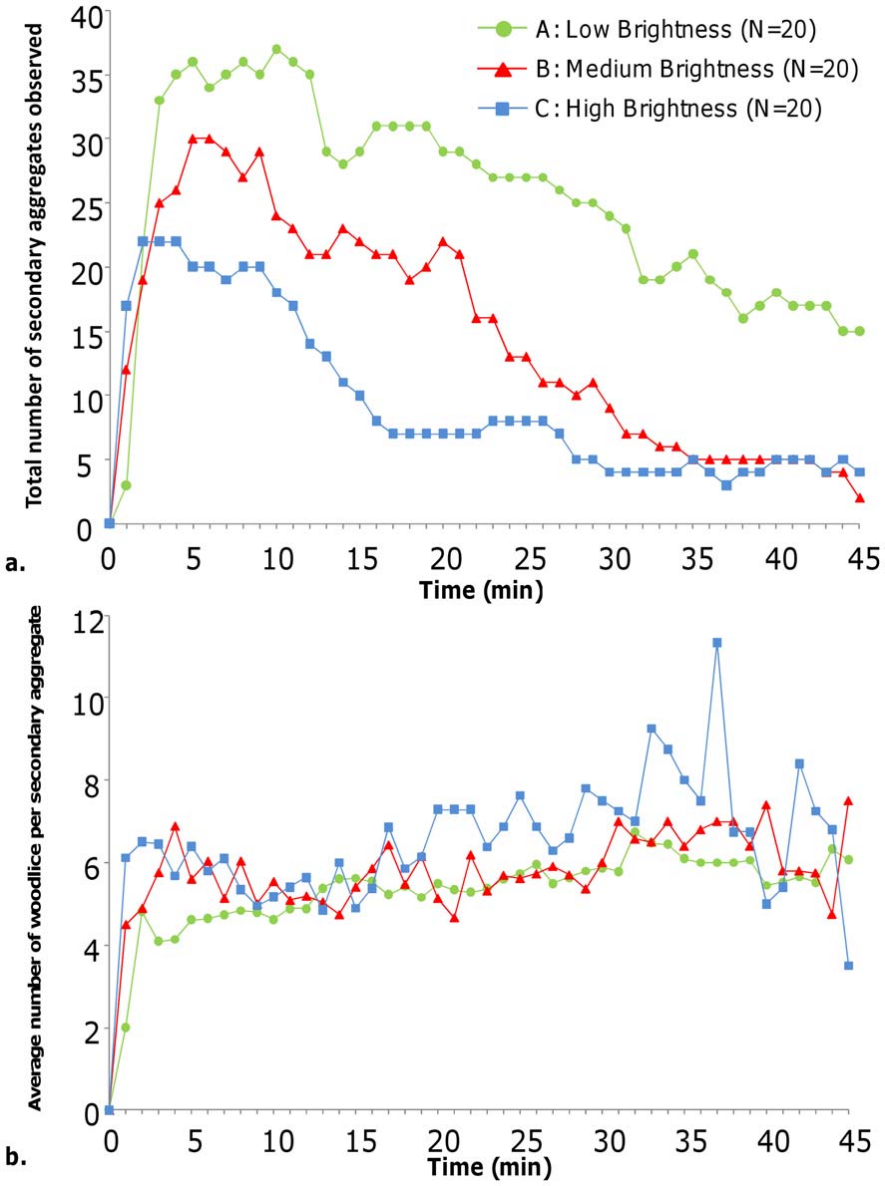
Dynamics of secondary aggregates. **a.** *Number of secondary aggregates observed during the experiments*. These numbers were observed in arenas under low, medium or high brightness corresponding to experimental conditions A, B and C. **b.** *Number of woodlice per aggregates.* Comparison between Low, medium and high brightness (respectively, experimental conditions A, B and C). No statistical differences were observed except for the 1^st^, 3^rd^ and 4^th^ minutes (A≠C, Kruskal-Wallis test followed by Dunn test, p<0.05).

**Figure 5:**
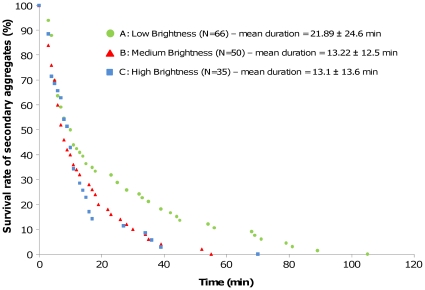
Survival rate of secondary aggregates observed in low, medium and high brightness (respectively, experimental conditions A, B and C). Total number of secondary aggregates observed and means duration of these aggregates are given in the legend of the figure for each experimental condition (means ± SD). Log Rank test showed significant difference between Low brightness and both other situations (p<0.05).

During the experiments, the evolution of the number of secondary aggregates was similar in every experimental condition: a quick increase was followed by a slow decrease (Figure 4a). Moreover, the average number of woodlice per secondary aggregates was relatively similar, being around 6 woodlice per aggregate whatever the experimental conditions (Figure 4b; differences can be observed between A and C at the beginning but it was not systematic). Hence during the experiments, the number of secondary aggregates was influenced by the aggregation process occurring in woodlice but not the number of woodlice per aggregate. Nevertheless, the evolution of the number of woodlice per aggregate was less regular in high brightness than in other condition. Furthermore, the survival of secondary aggregates was significantly higher in low brightness than in both other situations (Figure 5. Significant difference between low brightness and medium or high brightness, Log Rank test, Log rank statistic  = 5.4, p = 0.02 and 3.9, p = 0.047 for Low vs. Medium and Low vs. High brightness comparisons, respectively). The survival curves of secondary aggregates are well explained by exponential functions in every condition (y = 78.344.e^−0.038x^, R^2^ = 0.96; y = 92.199.e^−0.076x^, R^2^ = 0.99 and y = 96.197.e^−0.084x^, R^2^ = 0.95 for Low, Medium and High brightness). This result showed that the probability of disappearance of secondary aggregates is constant across time.

Finally, woodlice leaving a secondary aggregate were frequently observed walking in the arena in the high brightness whereas in low and medium brightness these woodlice had generally joined the final aggregate by the end of the experiments.

### Heterogeneous environment (conditions D, E, and F)

#### Woodlouse population outside shelters

**Figure 6:**
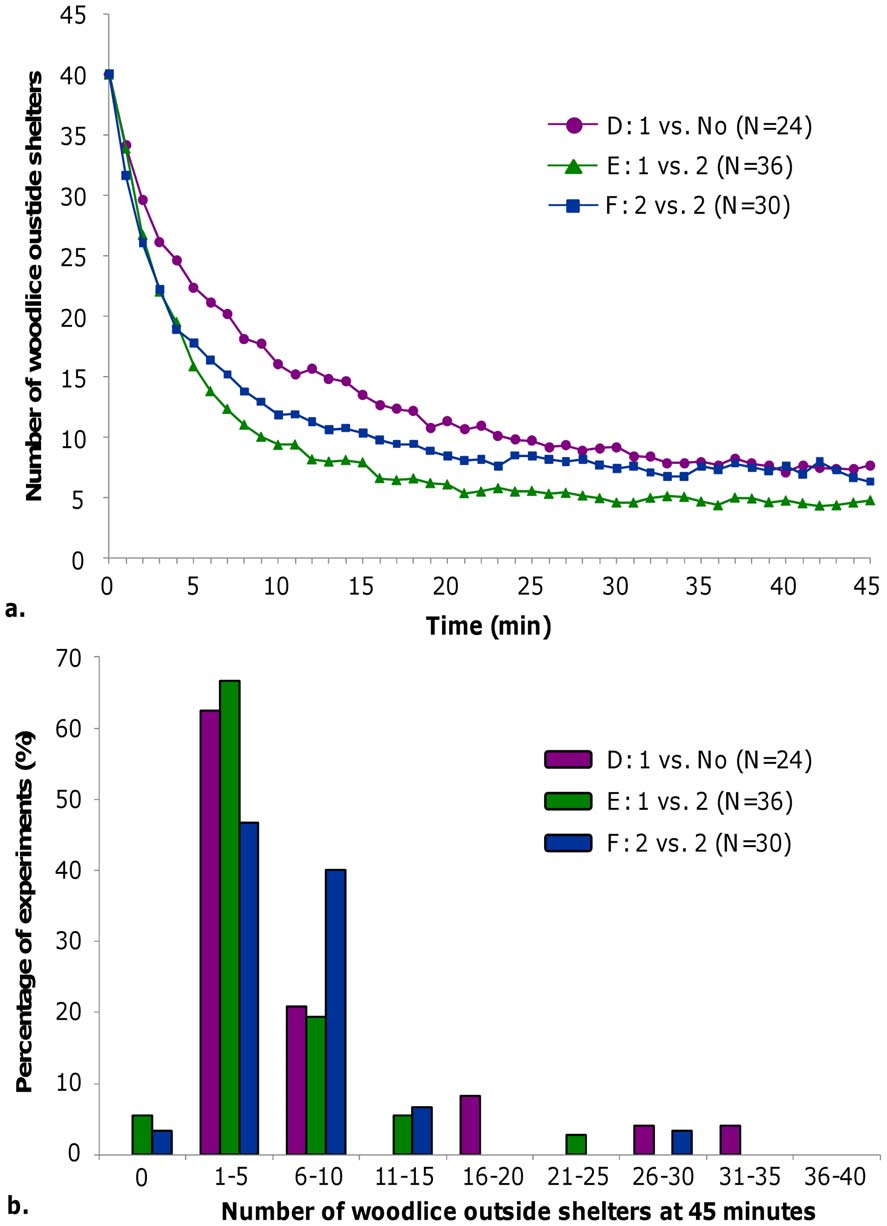
Dynamics of woodlice outside shelters. **a.** Evolution with time of the average number of woodlice outside shelters. **b.** Distribution of woodlice outside shelters at the end of experiments in heterogeneous conditions D, E, and F.

After release, the number of woodlice outside shelters exponentially decreased and at the end of experiments generally less than 10 woodlice were observed outside shelters (Figure 6a and example in Video S1). However, in four experiments (out of 90) more than 50% of woodlice were outside shelters at the end of experiments (Figure 6b). Among these four experiments, one (in condition F, 2 vs. 2 red filters) did not show any aggregation and three showed a peripheral main aggregate outside shelters. Despite these 4 experiments, aggregation under shelters was generally observed.

### Choice of a shelter

Whatever the experimental conditions more than 70% of experiments showed a selection of one of the two shelters (Table 1. No difference between conditions χ ^2^ test, χ ^2^ = 5.31, *d.f.* = 2, p = 0.07). Besides, in condition D, where there were a dark shelter and a bright one (without red filter), the selection of the dark shelter was significantly more frequent (Table 1; 88.9% of experiments; Fisher's exact test, p = 0.012). However, even if the bright shelter was rarely selected, it was in some cases (11.1% of experiments showed a bright choice; Table 1). The situation was different in conditions E where woodlice did not show any preference between the two dark shelters: even if selection of one of the two shelters was systematic (Table 1; 91.7% of experiments), the selection of the darkest one was only observed in 48.5% of experiments.

#### Dynamics of aggregation

**Figure 7:**
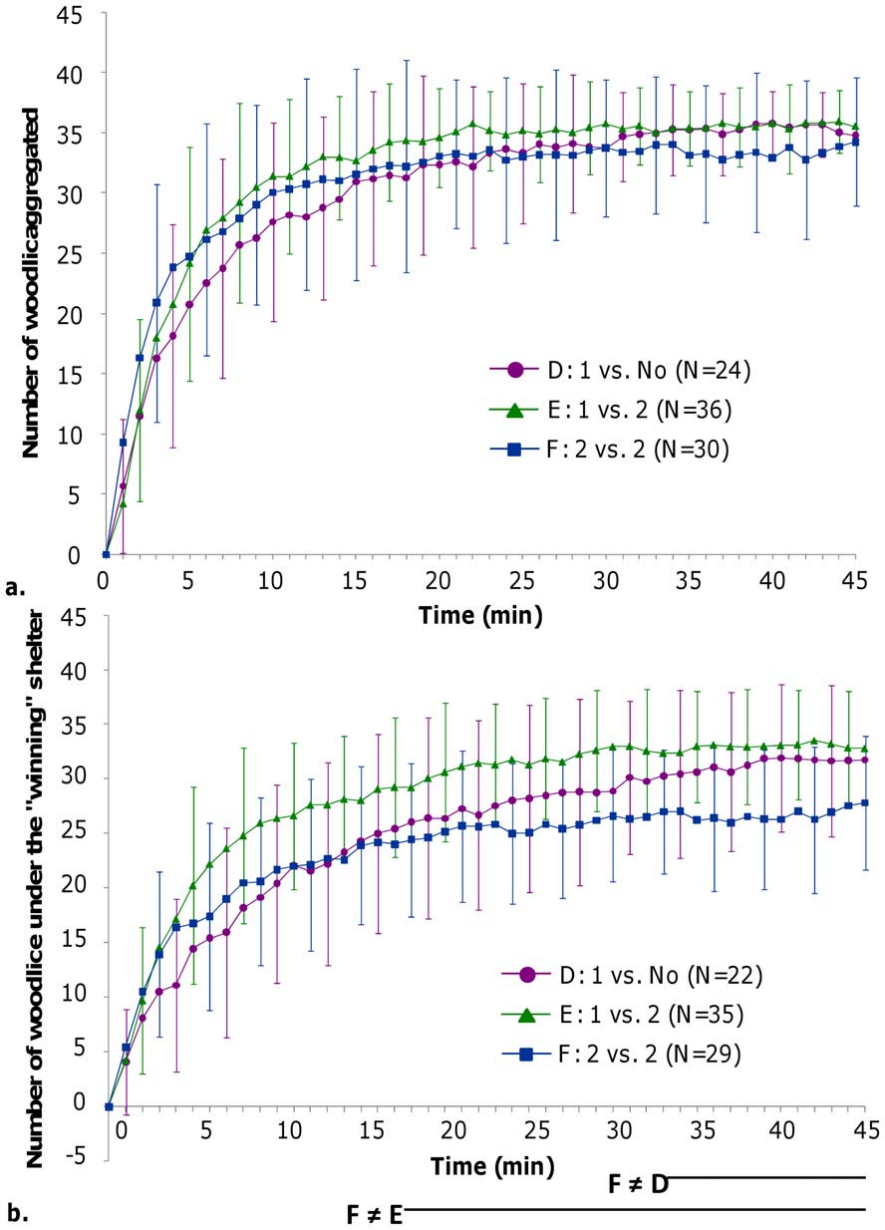
Comparison of the dynamics of aggregation, in heterogeneous set ups. Dynamics were observed in arenas corresponding to experimental conditions D, E, and F. ***a***: Total population of aggregated woodlice; ***b***: woodlice aggregated in the final aggregate under the “winning” shelter. Standard deviations are presented for each 3 minutes. The bottom part of graphic 6b represents the statistical differences obtained minute per minute using a Dunn's test, p<0.05; lines show differences.

The dynamics of aggregation in the heterogeneous environments were similar to those observed in the homogeneous ones with low or medium brightness. Indeed, soon after their release, in less than 5 minutes, global aggregation is observed (Figure 7a; no statistical differences between conditions D, E, and F; Kruskal-Wallis test, KW values<4.86; p>0.05). This aggregation is very stable since no difference was found in the total population of aggregated woodlice whatever the condition (Figure 7a; no difference between 15, 30, and 45 minutes for each of the conditions with shelters; Friedman's test, d.f. = 2, Fr = 4.4, 5.9 and 4.81 for D, E and F condition respectively, p>0.05).

**Figure 8:**
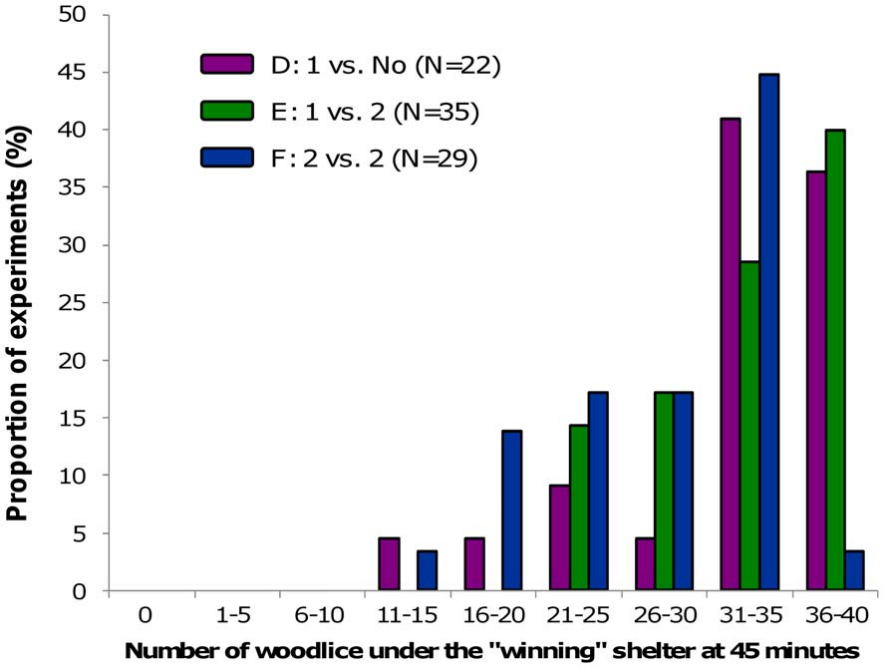
Distribution of woodlice in final aggregates in heterogeneous conditions D, E, and F.

Similarly, whatever the experimental condition, the number of woodlice in the final aggregate under the “winning” shelter quickly increased to reach more than 50% of woodlice in 10 minutes. At the end of experiments, this aggregate consisted of 25–30 woodlice on average (Figure 8). Even if at the beginning of the experiments the dynamics were strongly similar, the number of woodlice in the final aggregate was significantly lower in condition F compared to conditions E after 17 minutes and D after 33 minutes (Figure 7b; Kruskal-Wallis test, KW >7.23, p<0.05 followed by Dunn's test, p<0.05 from 17 to 45 minutes and p<0.05 from 33 to 45 minutes, respectively).

## Discussion

Since the seminal work of Allee [12], many studies have been concerned with woodlouse aggregation [13], [43], [49]. However, most focussed on its adaptive value and on the individual responses to environmental heterogeneity, and very little is known about the interaction between woodlice leading to aggregation, the signals or cues governing these interactions, and the dynamics of formation and stabilization of aggregates. However, the study of interaction between individuals during the formation of aggregates is required to better understand the observed aggregation patterns and their adaptive values [50], [9].

In our experiments, aggregation is a robust phenomenon. In most of the experiments, aggregations were observed and the dynamics of these aggregations were similar whatever the conditions and the location of the aggregates. Indeed, after a quick increase in the number of aggregated woodlice, this number stabilized at a high value. The absence of aggregation or a weak aggregation was observed in only six experiments out of the 150 carried out. This absence of aggregation was observed in high brightness without shelter and could result from the increase in activity with light intensity [51], [52], [15]. This increase in activity favoured the formation of small aggregates but also made the stabilization of these small aggregates more difficult. Indeed, the shorter duration of secondary aggregates and the high variability of the number of woodlice per secondary aggregate confirmed woodlice remained very active under these conditions. After this first aggregation, the instability caused by brightness could induce the progressive decrease in the number of woodlice in the small aggregates. As a consequence, at the end of experiments only one aggregate was observed and it contained most of the aggregated individuals. In contrast, the formation of the final aggregate in low and medium brightness, as well as in the environments with shelters, was progressive. Hence, even if the global aggregation showed that the total population of aggregated woodlice quickly increased, the growth of the final aggregate was progressive and also resulted from a relocation of woodlice previously aggregated in several secondary aggregates. Indeed, by leaving secondary aggregates, woodlice were found walking in the arena and could potentially integrate another aggregate. Finally, since the number of aggregates decreased with time, the woodlice generally left secondary aggregates to join the final one. In conclusion, light intensity had a weak influence on the speed of aggregation but could have some effects on the patterns and the stability of aggregation. A similar influence of light intensity on aggregation has been found in ants [53]. In the same way, the heterogeneities (shelters) do not influence the dynamics of aggregation but favour the stability of small aggregates.

Due to their thigmotactic behaviour [54], woodlice and aggregates were always observed at the periphery of the arena. Likewise, the location of aggregates was also influenced by light intensity, since in experiments with a choice between shelters, a dark shelter was chosen quasi-systematically by most woodlice. This is in accordance with their negative phototaxis [52], [22]. However, woodlice showed a preference for the dark shelter with one red filter (56 lux) rather than for the bright one without a filter (166 lux), but were unable to differentiate between two dark shelters varying in their light intensities (41 vs 56 lux). These last results contrast with the claim that woodlice are sensitive to low light intensity [55].

If the location of aggregates is influenced by thigmotaxis and negative phototaxis, these factors are not sufficient to explain the aggregation patterns observed. On the one hand, with only thigmotaxis, individuals should be randomly spaced along the periphery of the arena, and with only negative phototaxis, they should be equally distributed between shelters of identical darkness. Our results show that woodlice are not only tolerant of conspecifics but actually attract each other to constitute aggregates. Indeed, the selection of only one of two shelters in a binary choice (with identical shelters) and the aggregation in the homogeneous environment can only be explained by an inter-attraction between woodlice [50], [45]. Similar results have been extensively analysed in numerous subsocial or social insects (*e.g.* ants, spiders, cockroaches, caterpillars [48], [56]–[59]) and vertebrates [60]. Indeed, inter-attraction between conspecifics explains how cockroaches collectively choose a shelter or how sheep collectively forage. Indeed, the inter-attraction affects the probability of joining (or leaving) a shelter or an aggregate that increases (or decreases) with the aggregated population. Such modulations lead to the amplification of individual preference if the choices are not identical (*e.g.* shelters of different darkness) and mean that the collective response depends on the total population density.

The secondary aggregates and their dynamics observed in both the homogeneous environment and the heterogeneous one either under the second shelter or outside demonstrate social attraction between woodlice. Similar patterns and dynamics were observed in an ant cemetery in which clustering processes results from self-organizing dynamics ruled by local attraction [61]. Furthermore, the secondary aggregates and the observation of aggregation in less favourable places (such as under the bright shelter, which was rarely observed) show that inter-attraction can, to a certain extent, outweigh individual preferences.

These aggregations in unfavourable sites seem sub-optimal. On the one hand, secondary aggregates are less effective in the reduction of water loss [62]–[64]. On the other hand main aggregates could be observed under the bright shelter without a red filter while another dark shelter was available and known by woodlice. More investigations will be necessary to understand the benefits for woodlice of this inter-attraction which could induce potentially sub-optimal decisions. Such analyses are available in social species [65]. Indeed, in ants, some colonies could exploit poor or distant food sources while better or closer site are available [66]–[68]. It has been shown that a weak probability of making an error could improve the chance of discovery of better food sources even if it sometimes results in a sub-optimal response [69], [70]. In woodlice, the weak discrimination at the group level between shelter with one filter and shelter with two filters and the other sub-optimal responses could result from the high speed of collective decision which could trap the group in this first choice [71], [72]. A big aggregate has the disadvantage of increasing competition between individuals in the groups [73], [25]. In this respect, secondary aggregates could not be considered necessarily sub-optimal but could indirectly result from the trade-off between benefits and costs of the larger aggregate. Hence, the understanding of the adaptive value of secondary aggregates could be improved by using theories about social behaviour [74].

More experiments should be undertaken to decipher the signal used by woodlice in their inter-attraction. The role of aggregation pheromone coming from faeces has already been suggested [41], [42]. However, the high speed of aggregation shows that direct interactions play a great role in the dynamics. Hence, aggregation pheromones could only aid to stabilise the aggregates at longer time scales, whereas direct social interactions, perhaps mediated by other pheromones, could act at shorter time scales [75]. Pheromones acting at different temporal or spatial scales are well documented in social and gregarious insects (e.g. home-range or territorial marking and recruitment trails in ants [76], [77], [66], or marking with faeces and cuticular hydrocarbons in cockroaches [78]–[80]). Hence, different spatial or temporal scales could also be suggested in woodlice. Aggregation could result from local and distant attraction: volatile compounds could attract conspecifics (maybe due to gas ammonia [81]) and the stability of aggregates should be assured by other secretions (maybe from faeces). This scenario is in accordance with our results and the existing literature because aggregation was very fast, which corresponds to distant attraction, and aggregates were very stable. Moreover, theoretical knowledge of such decision-making systems allows us to predict that our results could be explained with thresholds (or quorums) which impact the entry or the exit of the shelter by individuals [45], [46], [82]. Hence, more analysis about individual behaviour and modeling will permit to decipher the content of social interactions and the rules underlying to the collective choice [83], [84].

To conclude, aggregation in woodlice results from a trade-off between individual preferences of woodlice (that is, in our case, being in darkness or at the periphery of the arena) and inter-attraction between individuals. Hence, it is possible to assume that the stability of an aggregate should depend on its location (being under a shelter) but also on the number of conspecifics.

Some woodlouse species, namely in the genus *Porcellio*, show subsocial behaviour, such as extended carrying of young in the marsupium, short- or long-term maternal provisioning, and biparental care with long-lasting family cohesion [85]. However, these species live in harsh environments [14]. Some other woodlouse species are not considered to be social; indeed, the only known social attribute related to their spatial distribution is the tolerance for conspecifics. Our results showed that in *Porcellio scaber*, the aggregation pattern is largely based on inter-attraction behaviour. Such phenomena are sensitive to the density of individuals and are an example of self-organization [50]. Aggregation is of particular interest because it is a prerequisite for the development of other forms of cooperation and could be a social step in the evolution of this clade [7]. In this respect some interesting comparisons can give new insights and direction for future research on woodlice. It has been demonstrated that as with woodlice, aggregation in cockroaches results from inter-attraction between conspecifics but is also partly influenced by individual preferences [79], [47], [86], [48]. Moreover, the benefits of aggregation in cockroaches are also reduction of their water loss and improvement of the transfer of bacteria between cockroaches [87]. Hence, such correspondences in mechanisms and adaptive values of aggregation in two different animals (crustacean and insect) allow us to envisage new research directions concerning a generic explanation of aggregation and a potential cascade of other social phenomena resulting from the local density increase [9]. These questions about the convergence of similar and simple mechanisms for different species are fundamental, not only for better understanding the mechanisms of organization, but also for making the link between the proximate and ultimate views of social evolution [88]. The consequence of such a generic logic could then be one of the keys to understanding the transition between different forms of cooperativeness and therefore different degrees of sociality.

## Supporting Information

Figure S1 **Radial distribution of woodlice density.**
(TIF)

Figure S2 **Angular distribution of woodlice.**
(TIF)

Video S1 **Example of recording.**
This is the record of a 2 versus 2 filters experiment (F condition – control) during the 20 first minutes. The video has been accelerated 10 times. Here, the woodlice select the left shelter in less than 5 minutes. After the first ten minutes, the aggregate stay stable during the following 35 minutes.
(MP4)

Text S1 **Determination of woodlice distribution in homogeneous set-ups.**
(DOC)
