# Supplementary figures and images for: Individual Preferences and Social Interactions Determine the Aggregation of Woodlice
Source: PLoS One. 2011 Feb 25;6(2):e17389. doi: 10.1371/journal.pone.0017389 (PMC3045452; doi:10.1371/journal.pone.0017389)

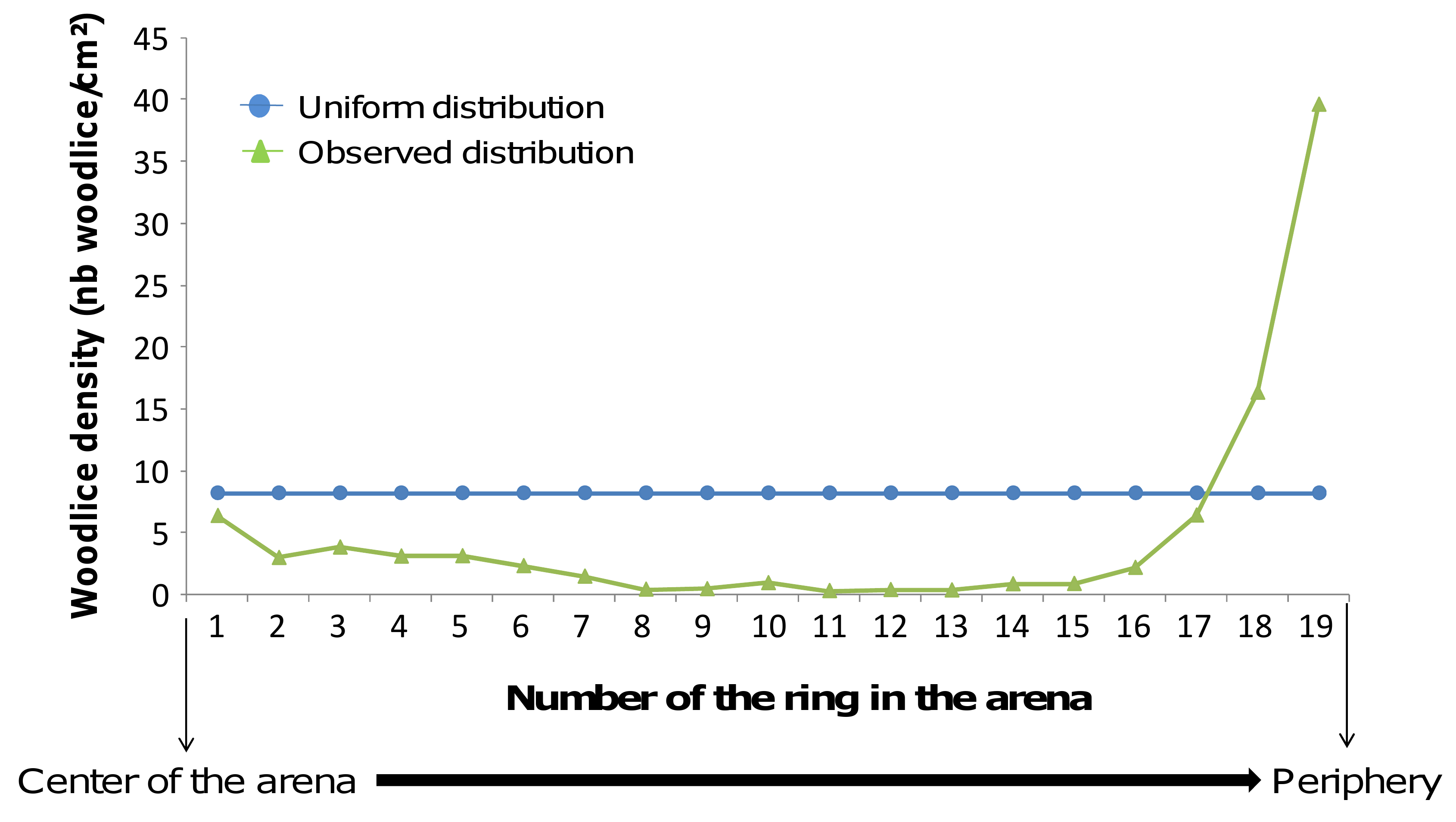

Supplement: Figure S1 — Radial distribution of woodlice density. (TIF) [file pone.0017389.s001.tif]

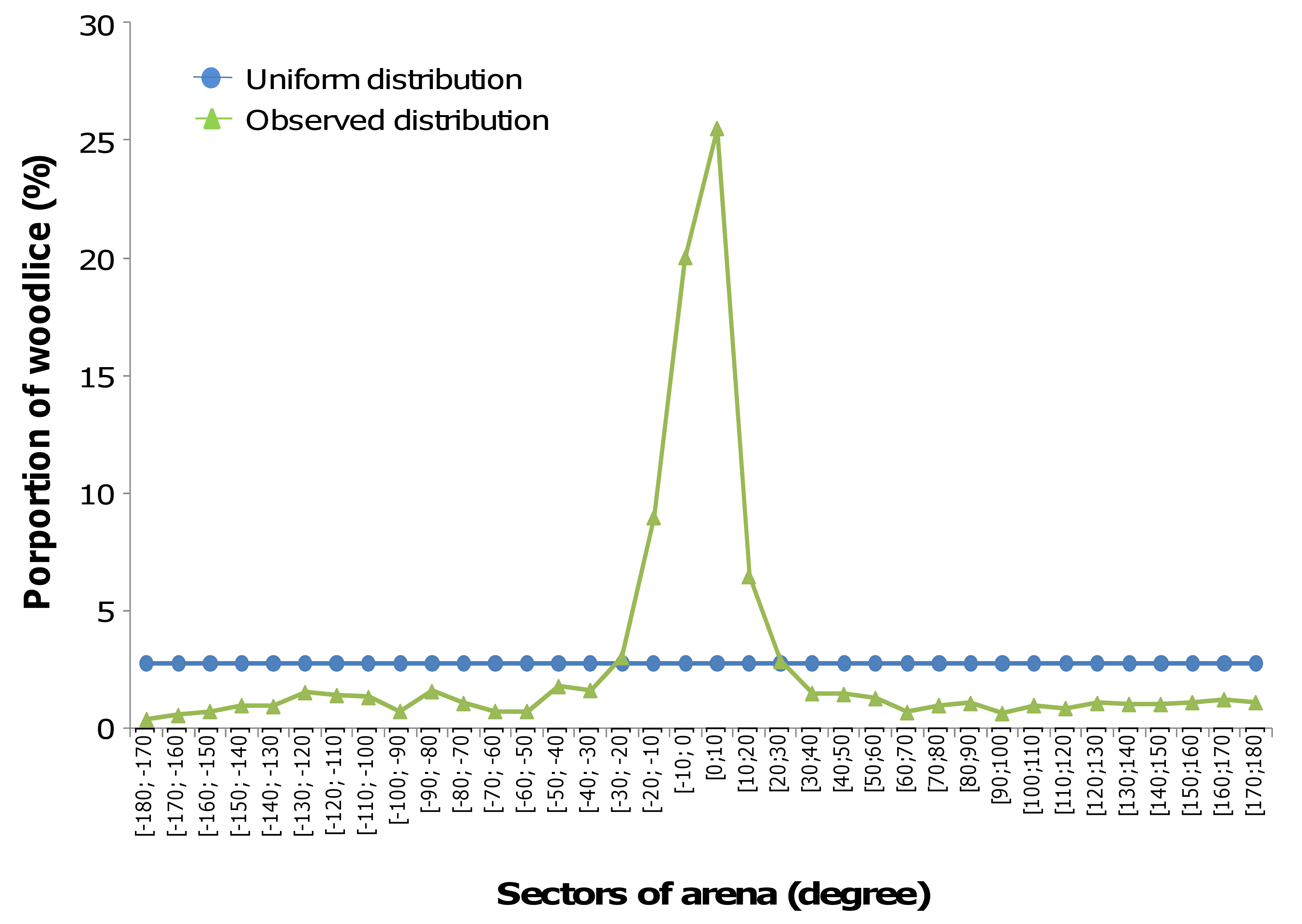

Supplement: Figure S2 — Angular distribution of woodlice. (TIF) [file pone.0017389.s002.tif]
