# Supplementary material for: Individual Preferences and Social Interactions Determine the Aggregation of Woodlice
Source: PLoS One. 2011 Feb 25;6(2):e17389. doi: 10.1371/journal.pone.0017389 (PMC3045452; doi:10.1371/journal.pone.0017389)
Supplement: Text S1 — Determination of woodlice distribution in homogeneous set-ups. (DOC) [file pone.0017389.s004.doc]

**Text S1. *Determination of woodlice distribution in homogeneous set-ups.***

In the homogeneous environment (where there were not preferential aggregation sites such as shelters), one problem was to define whether the distribution of woodlice corresponded to an uniform distribution or not. To do that, one mean is to compare the distribution of woodlice by using their polar coordinates. In polar coordinates, the uniform distribution is:

$$p(r,\theta)=\frac{2r}{2\pi R^{2}}\begin{matrix} & (1) \end{matrix}$$

where *p(r,θ)* is the probability to be at the distance *r* from the centre of the arena (radial coordinate) and at the angle θ (angular coordinate), *R* is the radius of the arena.

However, in our experiments, 90% of woodlice are observed at the periphery of the arena. Hence, we decided to separately analyse the radial distribution and the angular distribution. To do that, we compared our radial distribution and angular distribution experiment per experiment with the uniform theoretical distribution.

The theoretical uniform radial distribution is obtained thanks to the equation 2:

$$P(r)=\frac{2r}{R^{2}}\begin{matrix} & (2) \end{matrix}$$

Where *P(r)* is the probability to be at the distance r, *R* is the radius of the arena

We compared the theoretical distribution and the observed distribution thanks to a Kolmogorov-Smirnov goodness-of-fit test.

Besides, we tested the uniformity of our angular distribution thanks to a Rayleigh’s test [1].

We carried out these tests for each experiment: results are given in the paper.

**Results**

The radial and angular distributions observed were significantly different from the uniform distribution. Results of radial distribution showed that some woodlice could be found in the location of artificial central aggregation but most of the woodlice are located at the periphery of the arena. Such a distribution is not uniform (Figure S1). The Rayleigh tests showed that the angular distribution is not uniform. In summary, the woodlice are concentrated in an aggregate close to the periphery (Figure S2).

In Figures S1 & S2, results of global distribution of woodlice (N= 3x800 = 2400 woodlice) are given. For the graphic representation of angular distribution the peak of density (corresponding most often to the final aggregate) of each experiment was at θ = 0° of sector division of arena.
